# General and species-specific impacts of a neonicotinoid insecticide on the ovary development and feeding of wild bumblebee queens

**DOI:** 10.1098/rspb.2017.0123

**Published:** 2017-05-03

**Authors:** Gemma L. Baron, Nigel E. Raine, Mark J. F. Brown

**Affiliations:** 1 School of Biological Sciences, Royal Holloway University of London, Egham TW20 0EX, UK; 2 School of Environmental Sciences, University of Guelph, Guelph, Ontario, Canada N1G 2W1

**Keywords:** bumble bee queens, insect pollinator, insecticide toxicity, neonicotinoid insecticide, parasites, sublethal effects

## Abstract

Bumblebees are essential pollinators of crops and wild plants, but are in decline across the globe. Neonicotinoid pesticides have been implicated as a potential driver of these declines, but most of our evidence base comes from studies of a single species. There is an urgent need to understand whether such results can be generalized across a range of species. Here, we present results of a laboratory experiment testing the impacts of field-relevant doses (1.87–5.32 ppb) of the neonicotinoid thiamethoxam on spring-caught wild queens of four bumblebee species: *Bombus terrestris*, *B. lucorum*, *B. pratorum* and *B. pascuorum.* Two weeks of exposure to the higher concentration of thiamethoxam caused a reduction in feeding in two out of four species, suggesting species-specific anti-feedant, repellency or toxicity effects. The higher level of thiamethoxam exposure resulted in a reduction in the average length of terminal oocytes in queens of all four species. In addition to providing the first evidence for general effects of neonicotinoids on ovary development in multiple species of wild bumblebee queens, the discovery of species-specific effects on feeding has significant implications for current practices and policy for pesticide risk assessment and use.

## 1. Introduction

Pollination by wild insects, such as bees, is important for a vast array of crop systems and wild plants [1–4]. However, there is evidence for declines in wild bee populations since the 1900s on a global scale [5–12]. Pesticide use is one of several factors implicated in wild bee declines [13,14]. There is evidence from laboratory and field trials that both neonicotinoid and pyrethroid pesticides can have negative impacts on bumblebees at an individual level [15–21] and colony level [22–27]. Furthermore, pesticide exposure can impair the ability of bumblebees to pollinate effectively [28].

The growing body of research into neonicotinoid pesticide impacts on bumblebees generally focuses on *Bombus terrestris* as a model species within Europe and *B. impatiens* in North America. The ease of rearing these species in laboratory conditions, and their wide availability through commercial rearing facilities make them useful test organisms. However, there is considerable variation among bumblebee species in life-history traits, foraging behaviour and phenology, which may cause differences in their exposure and sensitivity to pesticides. Given these differences, extrapolating the effects of pesticides from one species to others is not always appropriate [29–34]. Neonicotinoids are one of the most widely used classes of pesticide in the world [35]. Residues have been found in the pollen and nectar of flowering crops as well as wild flowers in agricultural areas in the UK [36–37]. Several species of bumblebee are known to forage on crops such as oilseed rape [16,38–39] and field beans [1], as well as a range of wildflower species that naturally occur in agricultural areas. As such they are likely to be regularly exposed to low doses of pesticides [37]. Previous studies of non-neonicotinoid pesticides indicated variation in lethal dose among bumblebee species [40–42]. This variation in mortality may indicate that both lethal and sublethal impacts of neonicotinoids could also vary among species, although this has not been tested. As wild bumblebees are unlikely to be exposed to lethal doses (see above), here we investigate variation in sublethal impacts of the neonicotinoid insecticide thiamethoxam on four common bumblebee species, all of which are known to forage in agricultural areas. The four species selected—*B. terrestris*, *B. lucorum*, *B. pratorum* and *B. pascuorum*—differ in life-history and biological traits such as morphology, phenology and behaviour (electronic supplementary material, table S1).

Given the vital role of spring queens in maintaining bumblebee populations, we focused on assessing impacts at this stage in the life cycle. Queens of the four focal species were caught in the early spring, and exposed to a control, or one of two field-relevant doses of thiamethoxam. Thiamethoxam is one of three neonicotinoids currently under an EU moratorium for use on flowering, bee-attractive crops. It is widely used in the UK, for example, in 2015, 368 713 ha of land were treated with thiamethoxam [43]. Impacts of field-relevant exposure to thiamethoxam on feeding, survival, egg laying (colony initiation) and ovary development were assessed.

## 2. Material and methods

Queens of four bumblebee species, *B. terrestris*, *B. lucorum*, *B. pratorum* and *B. pascuorum*, were collected between March and April 2014, from Windsor Great Park, Surrey, UK (latitude: 51.417432 and longitude: −0.60481256). In total, 506 queens were collected (table 1). Species of the *B. lucorum* complex (*B. lucorum, B. cryptarum* and *B. magnus*) cannot be reliably separated using morphological features alone [44], but previous work has shown that only *B. lucorum* is present at our study site [45]. Individuals of each species were collected as early in the season as possible, and within a short time frame (electronic supplementary material, table S2). This minimized the time between emergence and capture, and as far as possible standardized the previous experience of individuals. Queens with stored pollen in their corbiculae were not collected as they were likely to have already established a nesting site.

**Table 1.** Summary of main results of impacts of three different doses of thiamethoxam (treatment) on life-history traits of four species of bumblebee queen. As infected and escapee queens were excluded from analyses, the total number of uninfected queens represents the individuals used for analyses. The average daily syrup consumption was calculated for uninfected queens only. The average terminal oocyte length was calculated for uninfected queens that survived the full four-week experiment. Averages presented are means ± s.e.

species	treatment	total collected	average thorax width (mm)	total uninfected	*n*, died	*n*, waxing	*n*, egg laying	*n* with oocytes	average oocyte length (mm)
*B. lucorum*	control	41	7.351 ± 0.028	12	3	3	3	9	3.103 ± 0.128
	low	39	7.360 ± 0.034	5	1	3	2	4	2.715 ± 0.140
	high	41	7.386 ± 0.035	10	1	7	1	10	2.849 ± 0.077
*B. pascuorum*	control	41	6.270 ± 0.043	17	3	7	3	13	1.850 ± 0.103
	low	41	6.330 ± 0.056	15	1	10	2	14	1.884 ± 0.094
	high	41	6.297 ± 0.038	16	1	6	1	12	1.594 ± 0.093
*B. pratorum*	control	38	6.250 ± 0.035	22	3	9	1	19	2.055 ± 0.074
	low	39	6.254 ± 0.036	15	1	10	0	14	1.785 ± 0.082
	high	39	6.290 ± 0.028	19	3	6	2	16	1.933 ± 0.089
*B. terrestris*	control	50	8.101 ± 0.034	35	2	11	7	32	2.915 ± 0.063
	low	48	8.042 ± 0.041	32	5	8	7	27	2.848 ± 0.083
	high	48	8.094 ± 0.033	32	3	8	6	27	2.780 ± 0.092

Several pesticides are used at the collection site: triticonazole (a fungicide) and acetamiprid (a neonicotinoid insecticide used for aphid control) are used as a treatment for roses (Roseclear Ultra formulation). These are applied between June and September, which means that while queens collected would not have been exposed in the spring, they may have had exposure the previous summer when emerging from their natal colonies. Windsor Park is surrounded by agricultural and urban areas, where queens may also have come into contact with pesticides used in gardens or crops. As such it was not possible to control for the prior pesticide exposure of queens collected, but as queens were randomly allocated to treatment groups (see below), it was assumed that any individuals with previous exposure would be randomly distributed.

Queen faecal samples were screened microscopically for the parasites *Crithidia bombi* (Trypanosomatidae)*, Nosema bombi* (Microsporidia), *Apicystis bombi* (Neogregarinida) and for *Sphaerularia bombi* (Nematoda) larvae using a Nikon eclipse (50i) compound microscope at 400× magnification. Only *C. bombi* was detected at this stage (*n* = 81), and infected queens were excluded from the experiment. Queens were then established in Perspex queen-rearing boxes (13.3 × 8 × 5.6 cm), kept in a dark room at a constant temperature of 28°C and 50% humidity, and provided with ad libitum 50% inverted sugar syrup solution (Ambrosia syrup, E H Thorne Ltd, Market Rasen, UK), from now on referred to as syrup, and pollen pellets (Koppert Ltd, Haverhill, UK).

### (a) Pesticide exposure

Queens were randomly allocated to one of three treatment groups: control, 1 ppb thiamethoxam (low dose) and 4 ppb thiamethoxam (high dose). These doses are within the range of thiamethoxam residues found in stored pollen and nectar in wild foraging bumblebee colonies [37,46], and from pollen and nectar collected from oilseed rape flowers and wildflowers [36,37]. Analytical standard thiamethoxam (Pestanal, Sigma-Aldrich) was mixed with Acetone (Fluka, Sigma-Aldrich) to give a stock solution of 100 mg ml^−1^. Aliquots of this stock were diluted with syrup to give the final concentrations. The volume of acetone used in the high dose was diluted in the same way, to provide a solvent control. Samples of treated syrup from two dates in the experiment were collected and analysed for thiamethoxam residues using LC–MS (Food and Environment Research Agency, Sand Hutton, York, UK). The average residues were 1.87 ppb ± 0.065 s.e. (low dose), and 5.32 ppb ± 0.579 s.e. (high dose). Control samples were also tested and found to contain trace amounts of thiamethoxam (0.063 ppb ± 0.018 s.e.). Pollen was ordered from a commercial bumblebee company (Koppert Ltd, Haverhill, UK) whose policy is to stock pollen free from pesticides, and thus we believe the pollen to be free from contaminants.

Queens were provided with the pesticide-treated syrup for 14 days. Oilseed rape (*Brassica napus*) typically flowers from early April in the UK, and the bloom period can last for three to six weeks. Queens establishing a nest in the spring would need to forage for at least four weeks (until first adult workers emerge). A two-week exposure period, therefore, represents a conservative exposure time. The weight of treated syrup consumed was measured to an accuracy of 0.1 g (once after 7 days, at which point freshly treated syrup was provided and again after 14 days). Average daily consumption during this period was then calculated. The average evaporation rate was measured by keeping 10 feeders in empty rearing boxes for a week, and calculating the weight of syrup lost during this time; syrup consumption data were then corrected for evaporation. Untreated syrup was provided ad libitum for the remainder of the experiment.

### (b) Monitoring

Following the pesticide exposure period, queens were observed for a further two weeks (four weeks in total), and checked daily for mortality, signs of waxing behaviour (wax is produced by queens as part of their natural nesting behaviour [47]) and egg laying. A four-week observation period was used in this experiment in order to assess any immediate impacts of pesticide exposure on queens and ovary development soon after exposure.

Queens that died during the experiment were frozen at −20°C. After four weeks, all remaining queens were frozen.

### (c) Dissection

At the end of the four-week experiment, all queens were dissected using a Nikon (SM2800) dissecting microscope at ×10–30 magnification. The abdomen contents were checked for internal mites (*Locustacaris buchneri)*, and adult and larval nematodes (*S. bombi*). A Nikon eclipse (50i) compound microscope at ×400 magnification was used to screen samples from the hindgut, malpighian tubules and fat body for the parasites *C. bombi*, *N. bombi* and *A. bombi.* Queens infected by at least one of these parasites at this stage (*n* = 235; electronic supplementary material, table S2 shows distribution across species and treatments) were excluded from the analysis. The presence of developing oocytes was also recorded, and the length of each terminal oocyte was measured using an ocular graticule (at ×20 magnification). Terminal oocyte length is the standard proxy measurement in bumblebee research to assess investment into ovarian development [48]. Thorax width was measured using digital calipers. Dissections were done blind with respect to the pesticide treatment group.

### (d) Analysis

We used the AIC-IT approach to analyse our data, as it enables more informative testing of null and alternative hypotheses, even under the conditions of a controlled experiment, as well as potentially more accurate estimates of effect sizes [49,50]. Models were constructed to test the impact of pesticide treatment on syrup consumption during the two-week treatment period, survival to the end of the four-week experiment, initiation of waxing, initiation of egg laying and average oocyte length. For each analysis, a model selection process was undertaken using the Akaike Information Criteria corrected for small sample sizes (AICc value) to evaluate the best-fitting model [51]. Fixed factors included *Treatment* (control, low or high), *Species* and size (which was adjusted for species differences by calculating the *Z*-score for each individual (SizeZ = (individual size − mean size for that species)/standard deviation for each species)). Owing to the inconsistent distribution of parasites across treatments and species (electronic supplementary material, table S2), it was not possible to incorporate parasitism into the statistical analyses. Models including individual fixed factors, and combinations of these, were compared against the null model (all candidate models for each analysis can be found in the electronic supplementary materials, tables S6–S12). Where more than one model was considered a good fit (within two AICc units of the optimal model), model averaging was undertaken [51]. Final models were verified graphically for fit and to ensure all assumptions had been met [52,53]. Interpretation of the importance of factors within the final models was based on the size of the estimate (the larger the estimate, the greater the effect size of that factor) and 95% confidence intervals (those which did not cross zero were considered reliable and important to the model). Where treatment effects were found, a *post hoc* Tukey's test was used to compare treatment groups.

Linear models were used to analyse data on the average daily syrup consumption. To detect any species-level differences that were not purely size-related, the average daily syrup consumption was corrected to control for bee size (syrup consumption/(thorax width)³), giving a measure of consumption per unit volume of bee (g mm^−^³). Model selection was undertaken as described above.

Survival was analysed both in terms of survival to the end of the experiment (28 days), using a binomial generalized linear model (GLM) with a log link, and also in terms of the timing of death using a Cox regression.

The presence or absence of waxing behaviour and egg laying within the four-week experiment were also analysed using binomial GLMs. A Cox regression was used for the timing of egg laying. Only data for queens surviving the whole experiment were used.

The average terminal oocyte length (corrected for species by using the *Z*-score as described above) was analysed using a linear model. Again, only data for queens surviving the whole experiment were used.

All analyses were performed in R (v. 3.1.1 [54]) using the survival [55] and multcomp [56] packages.

## 3. Results

A total of 506 queens were collected, of which 12 escaped during the course of the experiment. A further 235 were found during dissection to be infected with at least one of the following parasites: *C. bombi, A. bombi, N. bombi, S. bombi* or *L. buchneri*. The prevalence and distribution of the different parasites across hosts made it impossible to include parasites as covariates in the analysis, and so these queens were not included in further analyses. Twenty-nine queens had possible signs of infection and to be conservative these were also excluded from analyses. The distribution across treatment groups of these infected queens, and the remaining 230 that were included in the analyses, is shown in electronic supplementary material, table S2.

### (a) Syrup consumption

**Figure 1.**
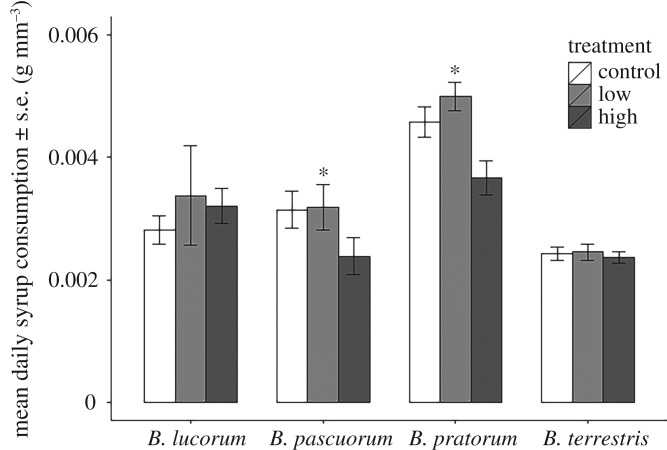
The average daily amount of syrup consumed by four species of bumblebee queen, treated with one of three thiamethoxam exposure scenarios (control, no pesticide; low, 1.87 ppb; high, 5.32 ppb). Bars show mean (±s.e.) consumption (grams) per unit volume of bee (cubic millimetres). An asterisk indicates a significant interaction (*p* < 0.05) between species and the high-dose treatment (electronic supplementary material, table S3).

**Figure 2.**
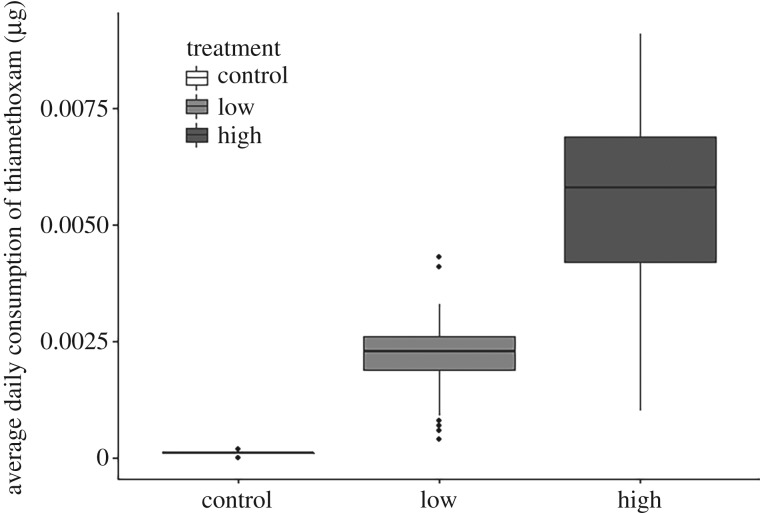
The average daily amount of thiamethoxam consumed by queens of four species of bumblebee. Values calculated from actual residue levels. Boxplots show the median (central line), interquartile range (box), range which lies within 1.5 times the interquartile range from the box (whiskers) and outliers (dots).

The high dose of pesticide treatment had a negative impact on syrup consumption by *B. pascuorum* (estimate = −0.00114, 95% CI [−0.00219, −0.0000973]) and *B. pratorum* (estimate = −0.001300, 95% CI [−0.00229, −0.00030]) queens (figure 1). The interaction between high dose and these species was important in the final model, but the treatment alone, and interactions with *B. terrestris* or *B. lucorum* were less important (electronic supplementary material, table S3). Despite the reduction in feeding by the queens in the high-dose group, the consumption of the active ingredient was still higher on average compared with the low and control groups (figure 2).

There were also species-level differences in syrup feeding, with *B. pratorum* consuming more syrup per cubic millimetres of body volume, compared with other species (estimate = 0.00159, 95% CI [0.00083, 0.00235]) (figure 1).

### (b) Ovary development

**Figure 3.**
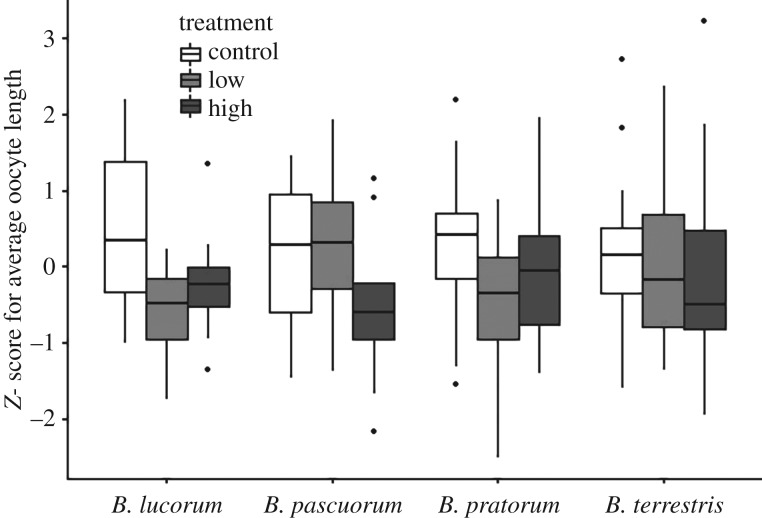
The relative oocyte length (*Z*-score for mean oocyte length) of four species of bumblebee queen after exposure to one of three thiamethoxam exposure scenarios (control, no pesticide; low, 1.87 ppb; high, 5.32 ppb). Boxplots show the median (central line), interquartile range (box), range which lies within 1.5 time of the interquartile range from the box (whiskers) and outliers (dots).

Exposure to the high dose of thiamethoxam caused a reduction in the length of terminal oocytes of queens. This was true across all species, and average oocyte length was reduced in queens from the high-treatment group by 8.1% (*B. lucorum*), 13.8% (*B. pascuorum*), 5.9% (*B. pratorum*) and 4.6% (*B. terrestris*), when compared with controls (table 1 and figure 3). As the high-pesticide treatment also caused a reduction in feeding, further analysis was undertaken to explore the influence of any effect this may have had on oocyte length. This involved further model selection including the amount of syrup consumed as a covariate (electronic supplementary material, table S5). In this case, treatment was still an important factor (estimate = −1.2518, 95% CI [−2.2882, −0.2155]), and queens in the high-dose group had significantly smaller oocytes compared with control and low groups (*p* < 0.05). The interaction term was also included in the final model, as was size, although these factors were less important (electronic supplementary material, table S5).

### (c) Survival

Across all species, 88% of queens (*n* = 203) survived for the four-week observation period. Pesticide treatment was not important for the overall survival rate of queens, or the time until their death.

Size (corrected for species using the *Z*-score) was an important factor in the binomial survival model (estimate = −0.655, 95% CI [−1.131, −0.178]) (electronic supplementary material, table S4); with queens that died during the experiment being slightly larger than average (electronic supplementary material, figure S1). The actual difference in size was fairly low (0.190 mm for *B. lucorum*, 0.135 mm for *B. pratorum*, 0.181 mm for *B. terrestris*) and *B. pascuorum* queens showed the opposite trend (surviving queens were on average 0.079 mm larger than those that died).

### (d) Waxing behaviour

Over half of the queens (53%) exhibited waxing behaviour during the experiment. There were species-level differences in the presence or absence of waxing (electronic supplementary material, table S4), but no treatment effects were detected.

### (e) Egg laying

There were differences in egg laying among species. More *B. terrestris* queens initiated a colony within four weeks than other species, and *B. pratorum* had the lowest colony initiation rate (table 1). Pesticide treatment was not included in the optimal models for egg laying, or the timing of egg laying.

## 4. Discussion

Wild bumblebee queens are likely to be exposed to pesticides while foraging or nesting in agricultural areas. This study provides the first evidence that field-relevant doses of thiamethoxam can have sublethal impacts on ovary development of queen bumblebees from multiple wild bumblebee species. Furthermore, species-level differences in response to pesticide exposure were observed; *B. pratorum* and *B. pascuorum* queens consumed less pesticide-treated syrup compared with controls, while no pesticide-induced reduction in feeding was observed for *B. lucorum* and *B. terrestris* queens.

Exposure to 5.32 ppb thiamethoxam in syrup resulted in a reduction in feeding by *B. pratorum* and *B. pascuorum* queens. No difference in feeding was found for *B. terrestris* and *B. lucorum*, suggesting that species may differ in their sensitivity to this compound. Previous species comparisons between honeybee and bumblebee workers [30], and between a bumblebee species and solitary bees [57], have found differences in sensitivity to another neonicotinoid, imidacloprid. Our results show that there are intra-generic differences among bumblebee species in response to sublethal doses of neonicotinoids. The mechanism behind the reduced feeding we observed could be related to a number of factors. Several pesticides have been reported to have a repellent effect on bees [58,59], which can result in a reduction in feeding. Alternatively, evidence for reduced feeding on imidacloprid-treated syrup by *B. terrestris* workers was suggested to be more likely due to toxicity rather than repellency, as the effect increased over time and with increasing dose [30]. Toxicity may cause bees to learn to avoid a substance that has an adverse effect [60], or may disrupt the physiological, behavioural or muscular processes involved in feeding [58]. However, both honeybees (*Apis mellifera*) and bumblebees (*B. terrestris*) appeared to prefer (rather than avoid) neonicotinoid-treated sugar water at nectar-relevant concentrations in laboratory choice tests [61]. Further testing is needed to elucidate the mechanisms controlling the change in feeding observed in this study, and why it differed across species.

Exposure to the high dose of thiamethoxam caused a reduction in the length of terminal oocytes of queens. However, we detected no impact of thiamethoxam exposure on egg laying by queens. Owing to the low number of queens that laid eggs during the experiment our power to detect potential impacts on this variable was low.

The inclusion of syrup consumption in the optimal model for oocyte length may indicate an interaction between syrup consumption and dose (although the confidence intervals suggest that this interaction term was less important than other factors in the model (electronic supplementary material, table S5)). Given that the high dose of pesticide caused a decrease in syrup consumption in some species, the resulting reduction in energy intake could be responsible for the impact on ovary development. However, despite the interaction term being controlled for, the high dose of pesticide remained an important factor in the model. Furthermore, species that showed no reduction in syrup feeding in response to pesticide exposure (*B. terrestris* and *B. lucorum*) had an equivalent reduction in oocyte length in the high-treatment group compared with controls (figure 3). These results suggest that a reduction in syrup feeding, caused by toxicity or repellency of the pesticide, cannot explain the treatment impact on oocyte development. One explanation could be that thiamethoxam impacts pollen consumption, as pollen contains essential nutrients for ovary development and brood production [62]. A reduction in untreated pollen consumption was observed in *B. terrestris* workers exposed to imidacloprid-contaminated syrup [19]. We were unable to measure pollen consumption, due to the waxing behaviour of queens, which made accurate measurement impossible. However, this would be an informative direction for further study. It is also possible that the metabolic cost of detoxification could lead to reallocation of nutrients, such as proteins, reducing nutrient availability for other biological processes (e.g. ovary development).

No effects on any of the traits measured were detected after exposure to the lower level of thiamethoxam used in this experiment (1.87 ppb ± 0.065 s.e.). This suggests that the impacts on feeding and oocyte development observed were dose-dependent. The two pesticide exposure levels used in the experiment are within the range of residues found both in wild foraging bumblebee colonies [37,46], and in pollen and nectar from flowering crops and wildflowers [36,37]. Queens of all four species tested in this experiment are known to forage on oilseed rape flowers and wildflowers in agricultural environments after emergence from hibernation [16], and as such are likely to be regularly exposed to these levels of pesticides. In fact, considering that exposure in the field is likely to occur via nectar and pollen [63], the doses used in this study could be considered conservative given that only the nectar was treated. Each of the species used in this experiment is likely to have a different exposure profile in the wild as a result of differences in foraging preferences, phenology and life-history traits (electronic supplementary material, table S2). For example, species with early-emerging queens, such as *B. pratorum* and *B. terrestris*, may only be exposed to pesticides in flowering crops in the second half of their foraging career, when nests have already been initiated. On the other hand, later-emerging queens, such as *B. pascuorum*, emerge when crops such as oilseed rape are in full flower, and so may have a higher likelihood of exposure if foraging in agricultural environments. Species-specific differences in phenology should be taken into account during pesticide risk assessments, and, if necessary, alternative forms of crop protection should be used at times when bumblebees and other wild species are most vulnerable.

No impacts of thiamethoxam exposure on survival were detected in this study. This supports previous findings that in the short term, exposure to a field-relevant dose of this neonicotinoid does not reduce survival in queens in the laboratory [16]. Other studies on the impacts of thiamethoxam on bumblebee queens have found reduced survival, but at a much later stage in the colony cycle [23], or at higher levels of pesticide exposure [64].

Queens with a detectable parasite infection were excluded from analysis in this study due to low levels of replication for each parasite within each species and across treatments. It would be interesting to further investigate the pesticide impacts on naturally parasitized queens, as negative interactions between parasites and pesticides have been observed in laboratory studies [23].

This study provides the first evidence that field-realistic exposure to thiamethoxam can have an impact on feeding and ovary development in multiple species of wild-caught bumblebee queens. Bumblebee queens are not currently considered in pesticide risk assessments for pollinators, and yet these results indicate that queens are sensitive to neonicotionoids in realistic exposure scenarios. Furthermore, differential sensitivity among species highlights the importance of considering the impacts of pesticides on a range of wild bee species. More information is urgently needed on residues and persistence of pesticides in crops, wild plants and in wild bee nests in order to accurately assess the exposure risks for the full range of species and castes of bees likely to encounter them. This is essential for understanding and managing the threat to wild bees from agrochemicals, and preventing further declines as a result of exposure to these pest control products.

## Supplementary Material

Baron et al Supplementary Figure, Tables, Analyses, R code

## Data accessibility

Data from this work have been uploaded to Dryad http://dx.doi.org/10.5061/dryad.h3c18 [65].
