# Supplementary material for: General and species-specific impacts of a neonicotinoid insecticide on the ovary development and feeding of wild bumblebee queens
Source: Proc Biol Sci. 2017 May 3;284(1854):20170123. doi: 10.1098/rspb.2017.0123 (PMC5443941; doi:10.1098/rspb.2017.0123)
Supplement: Baron et al Supplementary Figure, Tables, Analyses, R code [file rspb20170123supp1.pdf]

General and species-specific impacts of a neonicotinoid insecticide on the ovary development and feeding of wild bumblebee queens

Gemma L. Baron[1], Nigel E. Raine[1,2†], Mark J. F. Brown[1†]

[1] School of Biological Science, Royal Holloway University of London, Egham TW20 0EX
[2] School of Environmental Sciences, University of Guelph, Guelph, Ontario, N1G 2W1, Canada
[†] These authors contributed equally to this work

Electronic Supplementary Material

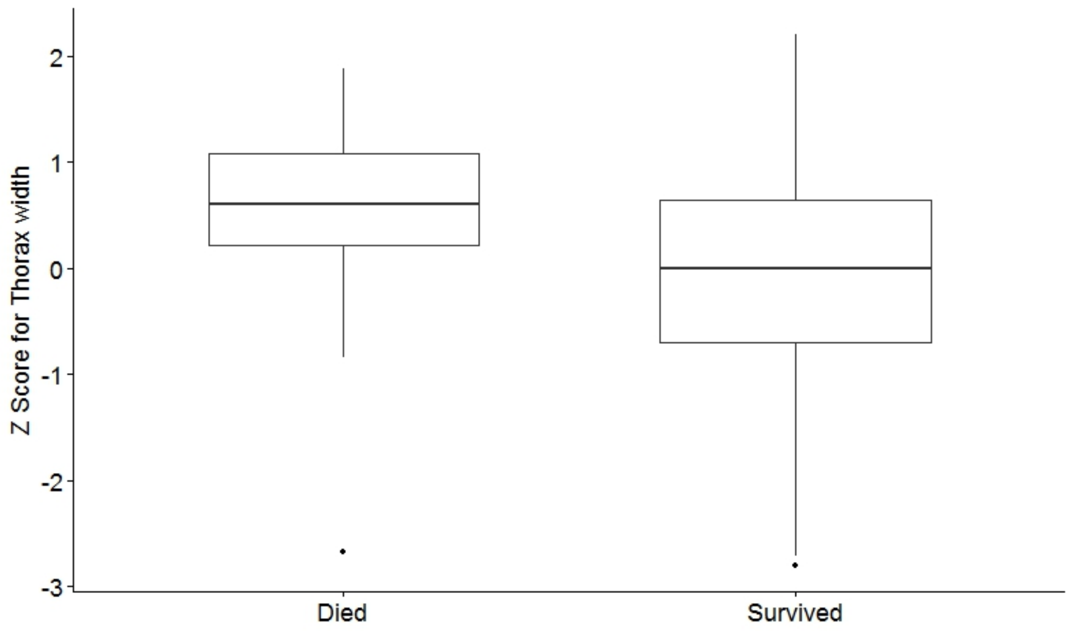

Figure S1: The relative size (Z score for thorax width) of bumblebee queens that died or survived during the four week experimental period. Boxplots show the median (central line), interquartile range (box), range which lies within 1.5 time of the interquartile range from the box (whiskers), and outliers (dots).

Species	Queen Size (length mm) [1]	Queens foraging [1,2]	Nesting sites [1]	Workers foraging [1]	Larval feeding strategy [1]	Colony size	Worker tongue length (mm) [7]	Worker foraging range (m) [8,9,10,11]
Bombus lucorum	18-21	March - April	Below-ground	Mid-April - July	Pollen storer	Medium (~100 workers) [3]	Short (7.5 ± 0.5)	No data available
Bombus pascuorum	15-18	April - May	Above ground - long tussocky grass	Late April - throughout summer	Pocket maker	Mid sized (100-200 workers) [4]	Long (8.5 ± 0.6)	312- 3200 [8, 9]
Bombus pratorum	15-17	Late Feb - March	Both below and above ground	Late March - late April	Pollen storer	Small (<100 workers) [5]	Mid (7.3 ± 0.4)	674 [8]
Bombus terrestris	20-23	Late Feb - April	Below-ground	March - June	Pollen storer	Large (several hundred workers) [6]	Short (7.6 ± 0.5)	583 – 3900 [8-11]

Table S1: Life-history traits of the four focal bumblebee species used in this study. Worker foraging ranges indicate estimates of the maximum ranges observed in several studies. Sources: [1] Alford (1975), [2] MJF Brown personal observations at collection site for this study, [3] Müller & Schmid-Hempel (1992), [4] Brian (1951; 1952), [5] Benton (2006), [6] Duchateau & Velthuis (1989), [7] Goulson *et al.* (2005), [8] Knight *et al.* (2009), [9] Chapman, Wang & Bourke (2003), [10] Wolf & Moritz (2008), [11] Osborne *et al.* (2008).

Species	Collection dates	Treatment	N	Average thorax width (mm) ± SE	Number of infected queens*					% Infected	N Uninfected
					C. bombi	***N. bombi***	***A. bombi***	***S. bombi***	***L. buchneri***		
B. lucorum	24 March - 11 April 2014	Control	41	7.351 ± 0.028	12		11	8		71	12
		Low	39	7.360 ± 0.034	14		7	17			5
		High	41	7.386 ± 0.035	11		6	15			10
B. pascuorum	9 - 17 April 2014	Control	41	6.270 ± 0.043	6	3	12	2		53	17
		Low	41	6.330 ± 0.056	8		10	3			15
		High	41	6.297 ± 0.038	7	1	12	3	1		16
B. pratorum	4 - 31 March 2014	Control	38	6.250 ± 0.035	4		1	1	7	39	22
		Low	39	6.254 ± 0.036	6		6	1	10		15
		High	39	6.290 ± 0.028	2	2	2	4	6		19
B. terrestris	11 - 12 March 2014	Control	50	8.101 ± 0.034	8		1	5		27	35
		Low	48	8.042 ± 0.041	7		2	5			32
		High	48	8.094 ± 0.033	10		1	4			32
**TOTAL**			**506**		**95**	**6**	**71**	**68**	**24**		**230**

Table S2: Summary of collection, treatment allocation, size, and infection status for the four focal species of queens used in this study.

*Totals Include queens with multiple parasite infections.

	Model	Fixed factors	Final model	Estimate	Standard error	95% CI	
						lower	upper
Syrup consumption (g/mm³)	lm	Treatment, Species	(Intercept)	2.94E-03	3.10E-04	2.34E-03	3.55E-03
			Treatment (Low)	4.28E-04	4.55E-04	-4.59E-04	1.31E-03
			Treatment (High)	1.31E-04	4.92E-04	-8.27E-04	1.09E-03
			Species (pasc)	1.34E-04	4.21E-04	-6.88E-04	9.56E-04
			**Species (prat)**	**1.59E-03**	**3.91E-04**	**8.29E-04**	**2.35E-03**
			Species (terr)	-5.03E-04	3.30E-04	-1.15E-03	1.41E-04
			Tment (Low) * Species (pasc)	-5.12E-04	6.31E-04	-1.74E-03	7.17E-04
			**Tment (High) * Species (pasc)**	**-1.14E-03**	**5.37E-04**	**-2.19E-03**	**-9.73E-05**
			Tment (Low) * Species (prat)	-1.36E-04	6.17E-04	-1.34E-03	1.07E-03
			**Tment (High) * Species (prat)**	**-1.30E-03**	**5.12E-04**	**-2.30E-03**	**-3.01E-04**
			Tment (Low) * Species (terr)	-5.32E-04	5.75E-04	-1.65E-03	5.90E-04
			Tment (High) * Species (terr)	-4.51E-04	4.76E-04	-1.38E-03	4.77E-04

Table S3: Summary of the linear model for average daily syrup consumption by four species of bumblebee queen. Syrup consumption was measured during a two week period when queens were exposed to one of two exposure levels (Low or High) of thiamethoxam or a control (Treatment). Consumption was adjusted to control for size of the individual by dividing the amount consumed (g) by volume of the thorax (mm^3). Fixed factors are those which were included in the model selection process. Final model details are from the composite model after model selection using the AICc (see Table S6 for candidate models). Factors highlighted in bold are those which were most important to the model, based on the size of the estimate and confidence intervals.

	Model	Fixed factors	Final model	Estimate	Standard error	95% CI	
						lower	upper
Survival	GLM	Treatment, Species, SizeZ	Intercept	2.17E+00	2.33E-01	1.71E+00	2.62E+00
			**SizeZ**	**-6.55E-01**	**2.45E-01**	**-1.13E+00**	**-1.78E-01**
	Cox regression	Treatment, Species, SizeZ	Null model				
Waxing	GLM	Treatment, Species, SizeZ	Intercept	1.28E+00	5.05E-01	2.96E-01	2.27E+00
			Species (pasc)	-1.14E+00	5.91E-01	-2.29E+00	1.11E-02
			Species (prat)	-9.93E-01	5.82E-01	-2.13E+00	1.42E-01
			**Species (terr)**	**-1.51E+00**	**5.49E-01**	**-2.58E+00**	**-4.38E-01**
Egg laying	GLM	Treatment, Species, SizeZ	(Intercept)	-1.03E+00	4.76E-01	-1.96E+00	-1.06E-01
			Species (pasc)	-7.90E-01	6.49E-01	-2.05E+00	4.75E-01
			**Species (prat)**	**-1.70E+00**	**7.63E-01**	**-3.18E+00**	**-2.09E-01**
			Species (terr)	-1.88E-01	5.39E-01	-1.24E+00	8.64E-01
			Size	1.72E-01	2.00E-01	-2.19E-01	5.62E-01
	Cox regression	Treatment, Species, SizeZ	Results as for GLM				

Table S4: Summary of models used for survival, waxing behaviour and egg laying in queens of four species of bumblebee. All analyses were performed across all species, on uninfected queens. Model indicates the type of model used (GLM is a generalised linear model). Fixed factors are those included in a model selection process (SizeZ is the z score for thorax width as described in the Methods). Final model details are from the optimal or composite model after model selection using the AICc (see Table S7-S10 for candidate models). Factors highlighted in bold are those which were most important to the model, based on the size of the estimate and confidence intervals.

	Model	Fixed factors	Final model	Estimate	Standard error	95% CI	
						lower	upper
Average Oocyte length	lm	Treatment, Species, SizeZ	(Intercept)	2.56E-01	1.14E-01	3.40E-02	4.78E-01
			Treatment (Low)	-3.20E-01	1.71E-01	-6.54E-01	1.32E-02
			**Treatment (High)**	**-4.56E-01**	**1.64E-01**	**-7.76E-01**	**-1.36E-01**
			SizeZ	1.32E-01	7.15E-02	-7.18E-03	2.72E-01
	lm	Treatment, Syrup, Species, SizeZ	(Intercept)	8.19E-02	3.94E-01	-6.87E-01	8.51E-01
			Treatment (Low)	4.01E-02	5.71E-01	-1.07E+00	1.15E+00
			**Treatment (High)**	**-1.25E+00**	**5.31E-01**	**-2.29E+00**	**-2.16E-01**
			Syrup	1.50E-01	3.27E-01	-4.87E-01	7.88E-01
			SizeZ	1.35E-01	6.97E-02	-8.73E-04	2.71E-01
			Tment (Low)*Syrup	-8.27E-02	4.76E-01	-1.01E+00	8.46E-01
			Tment (High)*Syrup	7.57E-01	4.54E-01	-1.29E-01	1.64E+00

Table S5: Summary of linear models used for the average terminal oocyte length of queens of four species of bumblebee (data were standardised for each species by using the Z score of Oocyte length as descibed in the Methods). Analyses include data from all four species, and include only queens that were uninfected and survived the full four week experiment. Fixed factors are those included in a model selection process using the AICc value (Syrup is the raw average daily syrup consumption data, SizeZ is the z score for thorax width as described in the Methods). Final model details are from the optimal or composite model after model selection using the AICc (see Tables S11, S12 for candidate models). Factors highlighted in bold are those that were most important to the model, based on the size of the estimate and confidence intervals.

Model Selection

Candidate models were constructed including each of the fixed factors individually and in biologically relevant combinations. These were compared with the null model (no fixed factors), and full model (all fixed factors). Two-way interactions between treatment and species were considered for linear models (Syrup consumption and Oocyte length), but not for generalised linear models (GLMs) due to small sample sizes for some groups. The AICc values were used (these were chosen over AIC values due to small sample sizes), and the optimal model (with the lowest AICc) was selected (highlighted in bold in each table below). When multiple models were within 2 AICc of the lowest (Delta ≤ 2), model averaging was undertaken (Johnson & Omland 2004).

Fixed Factors	Loglik	AICc	Delta	Weight
null	1200.994	-2397.9	108.96	0
Treatment	1203.634	-2399.1	107.81	0
Species	1254.680	-2499.1	7.81	0.013
**Treatment + Species**	**1260.062**	**-2505.6**	**1.28**	**0.34**
**Treatment * Species**	**1267.296**	**-2506.9**	**0**	**0.646**

Table S6: Candidate linear models for average daily syrup consumption (g/mm^3) by four species of bumblebee queen exposed to one of three thiamethoxam treatments.

Fixed Factors	Loglik	AICc	Delta	Weight
null	-83.190	168.4	6.13	0.036
Treatment	-83.074	172.3	9.99	0.005
Species	-82.571	173.3	11.05	0.003
**SizeZ**	**-79.107**	**162.3**	**0**	**0.774**
Treatment + SizeZ	-79.025	166.2	3.96	0.107
Treatment + Species	-82.439	177.3	14.99	0
Species + SizeZ	-78.459	167.2	4.92	0.066
Treatment + SizeZ + Species	-78.385	171.3	9.01	0.009

Table S7: Candidate GLMs for the survival to four weeks of four species of bumblebee queen exposed to one of three thiamethoxam treatments.

Fixed Factors	Loglik	AICc	Delta	Weight
**null**	**-1024.4**	**2048.8**	**0**	**0.547**
Treatment	-1024.38	2052.8	4.01	0.074
**Species**	**-1024.02**	**2050.0**	**1.25**	**0.292**
SizeZ	-1024.32	2054.8	5.96	0.028
Treatment + SizeZ	-1023.99	2054.1	5.30	0.039
Treatment + Species	-1023.92	2056.0	7.23	0.015
Species + SizeZ	-1024.30	2058.9	10.07	0.004
Treatment + SizeZ + Species	-1023.89	2060.2	11.37	0.002

Table S8: Candidate Cox regression models for the survival to four weeks of four species of bumblebee queen exposed to one of three thiamethoxam treatments.

Fixed Factors	Loglik	AICc	Delta	Weight
null	-140.292	282.6	3.14	0.095
Treatment	-139.610	285.3	5.87	0.024
**Species**	**-135.633**	**279.5**	**0**	**0.456**
SizeZ	-140.263	284.6	5.12	0.035
Treatment + SizeZ	-139.597	287.4	7.93	0.009
Treatment + Species	-134.550	281.5	2.06	0.163
Species + SizeZ	-135.618	281.5	2.07	0.162
Treatment + SizeZ + Species	-134.549	283.7	4.20	0.056

Table S9: Candidate GLMs for the presence or absence of waxing behaviour by queens of four bumblebee species exposed to one of three thiamethoxam treatments

Fixed Factors	Loglik	AICc	Delta	Weight
null	-93.318	188.7	2.40	0.132
Treatment	-93.035	192.2	5.93	0.023
**Species**	**-89.029**	**186.3**	**0**	**0.437**
SizeZ	-93.019	190.1	3.84	0.064
Treatment + SizeZ	-92.730	193.7	7.40	0.011
Treatment + Species	-88.683	189.8	3.54	0.075
Species + SizeZ	-88.653	187.6	1.35	0.222
Treatment + SizeZ + Species	-88.322	191.2	4.96	0.037

Table S10: Candidate GLMs for the presence or absence of egg laying by queens of four bumblebee species exposed to one of three thiamethoxam treatments. A cox regression analysis of timing of egg laying gave the same outcome – data not shown.

Fixed Factors	Loglik	AICc	Delta	Weight
null	-281.767	567.6	5.80	0.031
**Treatment**	**-277.522**	**563.2**	**1.45**	**0.275**
Species	-281.767	573.8	12.05	0.001
SizeZ	-279.744	454.6	3.82	0.084
**Treatment + SizeZ**	**-275.743**	**561.8**	**0**	**0.569**
Treatment + Species	-273.144	574.2	12.45	0.001
Species + SizeZ	-279.737	571.9	10.12	0.004
Treatment + SizeZ + Species	-275.734	568.2	6.43	0.023
Treatment * Species	-273.144	574.2	12.45	0.001

Table S11: Candidate linear models for the average length of oocytes of queens of four bumblebee species exposed to one of three thiamethoxam treatments.

Following from the model selection shown in Table S11, 3 additional models including an interaction between treatment and syrup consumption were added (Table S12 below).

Fixed Factors	Loglik	AICc	Delta	Weight
null	-281.767	567.6	8.28	0.007
Treatment	-277.522	563.2	3.93	0.066
Species	-281.767	573.8	14.53	0
SizeZ	-279.744	565.6	6.29	0.02
Treatment + SizeZ	-275.743	561.8	2.48	0.136
Treatment + Species	-277.508	569.6	10.28	0.003
Species + SizeZ	-279.737	571.9	12.59	0.001
Treatment + SizeZ + Species	-275.734	568.2	8.91	0.005
Treatment * Species	-273.144	574.2	14.93	0
**Treatment * Syrup**	**-273.207**	**561.0**	**1.68**	**0.202**
**Treatment * Syrup + SizeZ**	**-271.281**	**559.3**	**0**	**0.468**
Treatment * Syrup + SizeZ + Species	-269.593	562.6	3.27	0.091

Table S12: Candidate linear models for the average length of oocytes of queens of four bumblebee species exposed to one of three thiamethoxam treatments.

R Code:

The following R packages MuMin (Bartoń 2014), car (Fox & Weisburg 2011), survival (Therneau 2014), and coxme (Therneau 2015) were used for analysis

Syrup consumption LM

```
syr.lm<-lm(SyrupPerMM3~1, data=SQueen.Uninfect)
syr.lm.1<-lm(SyrupPerMM3~Treatment, data=SQueen.Uninfect)
syr.lm.2<-lm(SyrupPerMM3~Species, data=SQueen.Uninfect)
syr.lm.3<-lm(SyrupPerMM3~Treatment+Species, data=SQueen.Uninfect)
syr.lm.4<-lm(SyrupPerMM3~Treatment*Species, data=SQueen.Uninfect)
model.sel(syr.lm,syr.lm.1, syr.lm.2,syr.lm.3,syr.lm.4)
summary(syr.lm.4a)
summary(glht(syr.lm.4, mcp(Treatment="Tukey")))
Anova(syr.lm.4)
```

Survival GLM

```
surv.glm<-glm(Survival~1, binomial, data=SQueen.Uninfect)
surv.glm.1<-glm(Survival~Treatment, binomial, data=SQueen.Uninfect)
surv.glm.2<-glm(Survival~SizeZ, binomial, data=SQueen.Uninfect)
surv.glm.3<-glm(Survival~Species, binomial, data=SQueen.Uninfect)
surv.glm.4<-glm(Survival~Treatment+SizeZ, binomial, data=SQueen.Uninfect)
surv.glm.5<-glm(Survival~Treatment+Species, binomial, data=SQueen.Uninfect)
surv.glm.6<-glm(Survival~Species+SizeZ, binomial, data=SQueen.Uninfect)
surv.glm.7<-glm(Survival~Treatment+Species+SizeZ, binomial, data=SQueen.Uninfect)
model.sel(surv.glm, surv.glm.1,surv.glm.2,surv.glm.3,surv.glm.4,surv.glm.5,surv.glm.6,
    surv.glm.7)
summary(surv.glm.2)
```

Survival Cox Regression

```
surv.cox<-coxph(Surv(SurvivalTime, Death)~1, data=SQueen.Uninfect)
surv.cox.1<-coxph(Surv(SurvivalTime, Death)~Treatment, data=SQueen.Uninfect)
surv.cox.2<-coxph(Surv(SurvivalTime, Death)~SizeZ, data=SQueen.Uninfect)
surv.cox.3<-coxph(Surv(SurvivalTime, Death)~Species, data=SQueen.Uninfect)
surv.cox.4<-coxph(Surv(SurvivalTime, Death)~Treatment+SizeZ, data=SQueen.Uninfect)
surv.cox.5<-coxph(Surv(SurvivalTime, Death)~SizeZ+Species, data=SQueen.Uninfect)
surv.cox.6<-coxph(Surv(SurvivalTime, Death)~Treatment+Species, data=SQueen.Uninfect)
surv.cox.7<-coxph(Surv(SurvivalTime, Death)~Treatment+SizeZ+Species, data=SQueen.Uninfect)
model.sel(surv.cox,surv.cox.1,surv.cox.2,surv.cox.3,surv.cox.4,surv.cox.5,surv.cox.6,
    surv.cox.7)
```

Waxing GLM

```
wax.glm<-glm(Wax~1, binomial, data=SQueen.Uninfect.live)
wax.glm.1<-glm(Wax~Treatment, binomial, data=SQueen.Uninfect.live)
wax.glm.2<-glm(Wax~SizeZ, binomial, data=SQueen.Uninfect.live)
wax.glm.3<-glm(Wax~Species, binomial, data=SQueen.Uninfect.live)
wax.glm.4<-glm(Wax~Treatment+SizeZ, binomial, data=SQueen.Uninfect.live)
wax.glm.5<-glm(Wax~Treatment+Species, binomial, data=SQueen.Uninfect.live)
wax.glm.6<-glm(Wax~SizeZ+Species, binomial, data=SQueen.Uninfect.live)
wax.glm.7<-glm(Wax~Treatment+Species+SizeZ, binomial, data=SQueen.Uninfect.live)
model.sel(wax.glm,wax.glm.1,wax.glm.2,wax.glm.3,wax.glm.4,wax.glm.5,wax.glm.6,
    wax.glm.7)
```

Egg laying GLM

```
egg.glm<-glm(Egg.exc~1, binomial, data=SQueen.Uninfect.live)
egg.glm.1<-glm(Egg.exc~Treatment, binomial, data=SQueen.Uninfect.live)
egg.glm.2<-glm(Egg.exc~SizeZ, binomial, data=SQueen.Uninfect.live)
egg.glm.3<-glm(Egg.exc~Species, binomial, data=SQueen.Uninfect.live)
```

```
egg.glm.4<-glm(Egg.exc~Treatment+SizeZ, binomial, data=SQueen.Uninfect.live)
egg.glm.5<-glm(Egg.exc~Treatment+Species, binomial, data=SQueen.Uninfect.live)
egg.glm.6<-glm(Egg.exc~SizeZ+Species, binomial, data=SQueen.Uninfect.live)
egg.glm.7<-glm(Egg.exc~Treatment+Species+SizeZ, binomial, data=SQueen.Uninfect.live)
model.sel(egg.glm,egg.glm.1,egg.glm.2,egg.glm.3,egg.glm.4,egg.glm.5,egg.glm.6,egg.glm.7,egg.glm.4a)
```

Egg laying Cox Regression

```
egg.cox<-coxph(Surv(EggTime, EGG)~1, data=SQueen.Uninfect.live)
egg.cox.1<-coxph(Surv(EggTime, EGG)~Treatment, data=SQueen.Uninfect.live)
egg.cox.2<-coxph(Surv(EggTime, EGG)~SizeZ, data=SQueen.Uninfect.live)
egg.cox.3<-coxph(Surv(EggTime, EGG)~Species, data=SQueen.Uninfect.live)
egg.cox.4<-coxph(Surv(EggTime, EGG)~Treatment+SizeZ, data=SQueen.Uninfect.live)
egg.cox.5<-coxph(Surv(EggTime, EGG)~Treatment+Species, data=SQueen.Uninfect.live)
egg.cox.6<-coxph(Surv(EggTime, EGG)~Species+SizeZ, data=SQueen.Uninfect.live)
egg.cox.7<-coxph(Surv(EggTime, EGG)~Treatment+Species+SizeZ, data=SQueen.Uninfect.live)
model.sel(egg.cox,egg.cox.1,egg.cox.2,egg.cox.3,egg.cox.4,egg.cox.5,egg.cox.6,
    egg.cox.7)
```

Terminal Oocyte Length LM

```
ooc.lm.1<-lm(AvOocZ~1, data=SQueen.Uninfect.live)
ooc.lm.2<-lm(AvOocZ~Treatment, data=SQueen.Uninfect.live)
ooc.lm.3<-lm(AvOocZ~Species, data=SQueen.Uninfect.live)
ooc.lm.4<-lm(AvOocZ~SizeZ, data=SQueen.Uninfect.live)
ooc.lm.5<-lm(AvOocZ~Treatment+Species, data=SQueen.Uninfect.live)
ooc.lm.6<-lm(AvOocZ~Treatment*Species, data=SQueen.Uninfect.live)
ooc.lm.7<-lm(AvOocZ~SizeZ+Species, data=SQueen.Uninfect.live)
ooc.lm.8<-lm(AvOocZ~Treatment+SizeZ, data=SQueen.Uninfect.live)
ooc.lm.9<-lm(AvOocZ~Treatment+SizeZ+Species, data=SQueen.Uninfect.live)
ooc.lm.10<-lm(AvOocZ~Treatment*SyrupCon, data=SQueen.Uninfect.live)
```

```
ooc.lm.11<-lm(AvOocZ~Treatment*SyrupCon+SizeZ, data=SQueen.Uninfect.live)
ooc.lm.12<-lm(AvOocZ~Treatment*SyrupCon+SizeZ+Species, data=SQueen.Uninfect.live)
model.sel(ooc.lm.1, ooc.lm.2,ooc.lm.3,ooc.lm.4,ooc.lm.5,ooc.lm.6,
    ooc.lm.7, ooc.lm.8, ooc.lm.9, ooc.lm.10, ooc.lm.11, ooc.lm.12)
```

Supplementary Material Reference List

Alford D. (1975) Bumblebees. Davis-Poynter Ltd, London.

Bartoń K. (2014) Mumin: Multi-model inference

Benton T. (2006) Bumblebees. Collins New Naturalist Library, Harper Collins, London, UK.

Brian AD. (1951) Brood development in *Bombus agrorum* (Hym., Bombidae). *Entomologist's Monthly Magazine* **87** 207-12.

Brian AD. (1952) Division of labour and foraging in *Bombus agrorum* Fabricius. *Journal of Animal Ecology* **1** 223-40 (doi:10.2307/1959)

Chapman RE, Wang J, Bourk AFG. (2003) Genetic analysis of spatial foraging patterns and resource sharing in bumble bee pollinators. *Molecular Ecology* **12** 2801-2808 (doi:10.1046/j.1365-294X.2003.01957.x)

Duchateau MJ, Velthuis HHW. (1989) Ovarian development and egg-laying in workers of *Bombus terrestris*. *Entomologia Experimentalis Et Applicata* **51** 199-213 (doi:10.1111/j.1570-7458.1989.tb01231.x)

Fox J, Weisburg S. (2011) An [R] Companion to Applied Regression. Second Edition. Sage, Thousand Oaks CA

Goulson D, Hanley ME, Darvill B, Ellis JS, Knight ME. (2005) Causes of rarity in bumblebees. *Biological Conservation* **122** 1-8 (doi:10.1016/j.biocon.2004.06.017)

Johnson JB, Omland KS. (2004) Model selection in ecology and evolution. *Trends in Ecology and Evolution* **19**, 101-108 (doi:10.1016/j.tree.2003.10.013)

Knight ME, Osborne JL, Sanderson RA, Hale RJ, Martin AP, Goulson D. (2009) Bumblebee nest density and the scale of available forage in arable landscapes. *Insect Conservation and Diversity* **2** 116-124 (doi:10.1111/j.1752-4598.2009.00049.x)

Osborne JL, Martin AP, Carreck NL, Swain JL, Knight ME, Goulson D, Hale RJ, Sanderson RA. (2008) Bumblebee flight distances in relation to the forage landscape. *Journal of Animal Ecology* **77** 406-415 (doi:10.1111/j.1365-2656.2007.01333.x)

Müller CB, Schmid-Hempel P. (1992) Variation in life-history pattern in relation to worker mortality in the bumblebee, *Bombus lucorum*. *Functional Ecology* **6** 48-56 (doi:10.2307/2389770)

Therneau T. (2014) A package for survival analysis in s. R package version 2.37-7.

Therneau T. (2015) Mixed effect cox models. R package version 2.2-5.

Wolf S, Moritz RF. (2008) Foraging distance in *Bombus terrestris* L. (Hymenoptera: Apidae). *Apidologie* **39** 419-427 (doi:10.1051/apido:2008020)
